# Education at the frontier between tradition and innovation: challenges of an international initiative in breaking through

**DOI:** 10.3389/fsoc.2024.1393051

**Published:** 2024-11-22

**Authors:** Birce Altıok, Luisa Conti

**Affiliations:** ^1^Migration Research Center at Koç University, Koç University, Istanbul, Türkiye; ^2^Friedrich Schiller University of Jena, Jena, Germany

**Keywords:** transformative education, peer-learning, migrant students, e-learning platform, educational design, learning environment, lifelong learning, Turkish school system

## Abstract

In a rapidly evolving global landscape, education stands as a linchpin for navigating complex challenges and fostering sustainable development. This article delves into the transformative potential of education, with a particular focus on insights gleaned from the KIDS4ALLL project. Rooted in contrasting perspectives of education, the study emphasizes the need for a paradigm shift toward fostering critical thinking, creativity, and inclusivity. The European Commission’s commitment to transformative education finds expression in initiatives like KIDS4ALLL, which harnesses digital platforms to prioritize peer learning and bridge divides. Through an exploration of reactions within Turkish schools to the project’s introduction, this study sheds light on the dynamics of change and resistance, offering valuable insights into the challenges and opportunities towards educational transformation. Drawing on empirical data from the project’s pilot phase, the study identifies key factors shaping the realization of educational change. By elucidating these factors, the article contributes to a nuanced understanding of transformative education, paving the way for informed strategies aimed at fostering inclusive, sustainable, and impactful educational practices on a global scale.

## 1 At the crossroad between past and future: where we are and where we head to

Education, as pointed out by Craft (1984) and critically examined by various authors (cfr. Bass and Good, 2004), is a multifaceted concept shaped by two intersecting perspectives. The first one traces education back to the Latin term “educare,” meaning molding and training. In this view, education is seen as the transmission of established values and knowledge from one generation to the next, with the goal of preservation. The second perspective, rooted in the Latin term “educere,” interprets education as a process of unfolding and leading out. This perspective envisions education as a means to let individuals’ potential flourish, fostering creativity and critical thinking to promote development and tackle contemporary challenges (Bass and Good, 2004, p.162). The second meaning becomes more and more relevant today, as the global society is going through deep changes which make it necessary to gird new generations with an absolutely new set of knowledge and skills and to help them to develop specific attitudes consciously. This is important for them as individuals—who must be able to fulfill their needs—and for them as citizens—who do not endanger others and have at best a positive impact on their communities.

Understanding education as a process shaping critical and creative agents rather than uncritical ‘reproducers’ is of existential importance today. This consideration is particularly poignant in light of the fact that dominant contemporary cultures all over the globe are rooted in or influenced by capital oriented economic structures which inevitably are leading to resource depletion and the destruction of the bases of human existence, reaching a point of no return and triggering multiple long-term crises (Lange, 2023). It is therefore of fundamental importance that new generations are free and are stimulated to create new, sustainable cultures (UN General Assembly, 2023, p. 3, 8; Hammond, 2021). In this historical context, education takes on the imperative role of being transformative. It should offer an experience that goes beyond imparting knowledge, actively shaping individuals who possess the capacity to consciously transform the reality they live in. A fundamental goal of education today is thus to foster the emergence of a socially and ecologically sustainable society (Bamber, 2019). The specific prescription of the seventh target of the fourth Sustainable Development Goal dedicated to inclusive and equitable quality education for all [UN (United Nations General Assembly), 2015, p.17] and to which 191 UN member states have committed, is as follows:

“By 2030, ensure that all learners acquire the knowledge and skills needed to promote sustainable development, including, among others, through education for sustainable development and sustainable lifestyles, human rights, gender equality, promotion of a culture of peace and non-violence, global citizenship and appreciation of cultural diversity and of culture’s contribution to sustainable development” [UN (United Nations General Assembly), 2015, p. 17].

The acknowledgment of transformative education as a fundamental tool for achieving a global sustainable shift is now officially recognized. Within this context, the European Commission has taken strides in supporting projects aimed at fostering innovation in formal, non-formal, and informal education. These initiatives often involve collaborative efforts between scientific and educational institutions. The outcomes of these projects typically include valuable suggestions for policy makers, contributing to the—at least potential—development of informed and effective strategies. Additionally, a key focus is on strengthening networks among diverse actors at the local level, fostering collaboration and synergy.

The investments made in transformative education extend beyond achieving the significant milestone by 2030, the targeted deadline of the Sustainable Development Goals (SDGs). They also serve as a response to the global autocratic-authoritarian trend that poses a threat to the social cohesion of plural societies. By implementing transformative education, efforts aim to counteract this drift and contribute to a more sustainable and dialogic global community. The strategy “Learners First” (2024–2030), endorsed at the last European Standing Conference of Ministers of Education “The Transformative Power of Education: Universal Values and Civic Renewal” aims specifically at the promotion of “a culture of democratic participation among all learners in order to safeguard democracy” (COE, 2023a).

These efforts, promoted by international institutions to democratize education and shape citizens capable of confronting permacrisis constructively face a dual challenge. The first hurdle lies in the inherent difficulty of altering deeply ingrained structures and processes. The second challenge arises from the reluctance of governments, in particular the conservative ones whose extremist, anti-democratic factions are gaining popularity, to endorse such transformation (Gottenhuber and Mulholland, 2020; Kroll and Zipperer, 2020).

Currently, many countries find themselves at a crossroads where progressive and conservative cultures intersect, potentially leading to clashes that impede a cultural shift toward the realization of the core principles of democracy which are human dignity, justice, equality, solidarity, peace, and freedom (Haarmann et al., 2020, p. 1). These values, crucial for fostering coexistence, are not innate but instead socially constructed (Arendt, 1994, p. 241); they are learned through socialization (Scherr, 2016, pp. 36–39). While legal frameworks provide a context for value dissemination, they alone cannot anchor them. Individual life experiences play a pivotal role in shaping values (Scherr, 2016, p. 37): What values are individuals exposed to? Which values are exemplified in their living environment and by their role models? Which ones are explicitly or implicitly promoted?

The development of democratic values is thus not an automatic outcome of a democratic system; rather, it is a deliberate process facilitated by the community (Scherr, 2016). Schools in democratic systems should play a crucial role in shaping individuals’ values and nurturing their democratic mindset (Dewey, 1916). This responsibility gains heightened significance in the present context, where urgent global challenges require citizens capable of collaborative and coordinated efforts while digitalization divides them (Lenehan, 2022, p. 6–7), primarily due to polarizing algorithms and information bubbles, representing a threat at a local, national and supranational level (Maati et al., 2022; Pira, 2023). Nevertheless, the digital transformation has also opened innovative opportunities for fostering individual and collective well-being, protecting human rights, promoting democracy, and advancing environmental sustainability (COE, 2023b, p. 10). In order to address the risks posed by digitality, leveraging its potential for positive change becomes essential in promoting resilience and inclusion.

Key Inclusive Development Strategies for a LifeLong Learning (KIDS4ALLL), the project at the center of this special issue of Frontiers in Human Dynamics, is specifically designed to address this challenge. It adopts a strategy explicitly prioritized by the latest aforementioned Council of Europe Standing Conference of Ministers of Education: the creation of an appropriate digital platform for promoting peer learning (COE, 2023a,b, p. 7). This initiative serves a dual purpose: combating exclusion, enhancing competences and empowering users—both students and educators—to become transformative agents.

This article aims to present the reactions to the introduction of the platform in the existing system and observe the dynamic between change and resistance to it. This exploration is crucial to stimulate reflection on the opportunities and limitations of turning innovation actions into a long-term and broader change. To provide a more in-depth understanding, this paper confines its analysis to the pilot phase of the project implemented in schools in Turkey. It is noteworthy to mention that Turkey does not represent an anomalous case; instead, the experiences documented here resonate with those observed in other countries, as indicated in various articles within this special issue and additional publications (e.g., Petropoulos et al., 2023).

After describing the specificity of this international innovation action and highlighting its inclusive principles in the next section (2), we will describe the context of the study and the empirical instruments applied (section 3). The data collected, presented in four categories (section 4), will be discussed in a final chapter in which we will identify factors with major impact on the realization of educational change (section 5).

## 2 KIDS4ALLL key strategies toward pedagogical innovation

Funded by the European Commission in the context of the Horizon 2020 program “Europe in a changing world—inclusive, innovative, and reflective societies” Key Inclusive Development Strategies for a LifeLong Learning (KIDS4ALLL) is one of the selected research and innovation actions toward “Mapping and overcoming integration challenges for migrant children” (COE, 2020). This project aims at developing a multilingual learning environment through the promotion of peer learning within formal, non-formal, and informal educational settings in order to overcome isolation and at the same time to foster the development of key competences. The learning approach employed by KIDS4ALLL encompasses three key components: (1) knowledge acquisition, (2) skills training, and (3) attitude transfer, facilitating the development of comprehensive lifelong learning competences through collaborative and co-creative learning processes. Tailored to address the educational requirements of children, especially migrant children, and educators serving as guides for transformative lifelong and lifewide learning, the project is anchored in three Key Inclusive Development Strategies (KIDS) for LifeLongLearning (LLL). These strategies constitute the project’s specific objectives: (1) cultivating competences; (2) enhancing educators’ methodological competences for inclusive and participatory teaching, training, and intercultural dialogue; and (3) experimenting with the concept of peer-to-peer learning through buddyship collaboration (guided pairing of learners). The learning method is supported by online and offline tools constituting the KIDS4ALLL learning environment: an e-learning platform, a handbook, which is a compact version of the platform on paper, and an app for creating digital postcards to share on the platform.

The contents for students revolve around the eight key competences for lifelong learning, as endorsed by the Council of the European Union in 2019. These competencies include literacy, multilingualism, cultural awareness and expression, citizenship, personal, social and learning to learn, digital, mathematical and competence in science, technology, and engineering, and entrepreneurship. These competences are deemed “essential to citizens for personal fulfillment, a healthy and sustainable lifestyle, employability, active citizenship, and social inclusion” (COE, 2019, p.4). They are defined as a combination of knowledge, skills, and attitudes that can be cultivated throughout one’s life in formal, non-formal, and informal educational contexts. Conversely, the contents tailored for teachers concentrate on three key competences: dialogic and intercultural competence, collaborative learning, and socio-emotional skills and global competence. These competences have been specifically chosen for their relevance in aiding teachers to fulfill their responsibilities in an inclusive manner. The platform’s contents, coupled with its dialogic approach on which its design roots, actively prompt users to reflect on the contents and transfer them into their lifeworld. This approach nurtures autonomous thinking, critical reflection, and the embodiment of values and attitudes as well as the development of skills and knowledge crucial to a democratic society.

Key Inclusive Development Strategies for a LifeLong Learning embodies a collaborative endeavor of an international team of researchers and educators from eight EU countries and three non-EU countries dedicated to creating in dialogue with civil society organizations and policymakers a universally applicable concept that easily integrates into diverse learning environments, encompassing both formal and informal settings across different countries and contexts. The promising strategy behind its design lies in a negotiated standardization, a process carefully tailored to ensure widespread acceptance in diverse contexts. This has meant making strategic decisions, such as excluding certain sensitive topics from the contents and limiting the visual representation of diversity in the tutorials in order to be allowed in different regions and institutions. So for instance, in the video tutorials, where there is an effort to represent people in their diversity, explicitly non-heteronormative individuals and girls wearing crop tops have been substituted with other characters.

The localization of the contents, a key aspect of KIDS4ALLL, is achieved through its dialogic approach which recognizes learners as “epistemic authorities” (Baraldi et al., 2022) and allows them to share their valuable knowledge and to extend it, seeking new information and developing new ideas. In this way, the users are stimulated to exchange on a certain topic and to share and seek knowledge on that topic in their context, e.g., how the school system is structured. This does not only foster a sense of ownership and engagement but also ensures that the initiative resonates authentically within diverse cultural and regional contexts.

The principle of multilingualism is integral to KIDS4ALLL. With outputs available in 14 languages, the initiative ensures widespread accessibility for educational purposes across numerous countries and particular benefits for young people on the move. The possibility to enjoy the same content in various languages not only emphasizes the significance of equal access to knowledge irrespective of language proficiency but also serves as a catalyst for fostering interest in other languages. The multilingual approach addresses language barriers and enhances inclusivity, promoting a learning environment that transcends linguistic limitations. Moreover, it also provides individuals with the opportunity to broaden their linguistic skills and advance their understanding across diverse cultural and linguistic landscapes.

The commitment to multilingualism within KIDS4ALLL aligns with its overarching ideals of promoting a sustainable society. This dedication extends to decisions that prioritize privacy and resist the use of commercial platforms associated with big companies, thereby avoiding advertising and potential data security concerns. By consciously choosing not to rely on external services and platforms, KIDS4ALLL increases its suitability for educational institutions. The avoidance of commercial sites, especially those outside the EU, aligns with the privacy policies of many schools. Consequently, KIDS4ALLL ensures a secure and privacy-conscious space, making it an ideal choice for educational purposes and contributing to the realization of its mission in fostering a safe and compliant digital environment for learners worldwide. This commitment imposes though limitations which result for example in a relatively small number of videos. KIDS4ALLL addresses these restrictions with innovative solutions. For instance, it incorporates visuals without words and when it employs short videos in English, it integrates them with explanatory files, which include screenshots and summaries in different languages.

**Figure 1:**
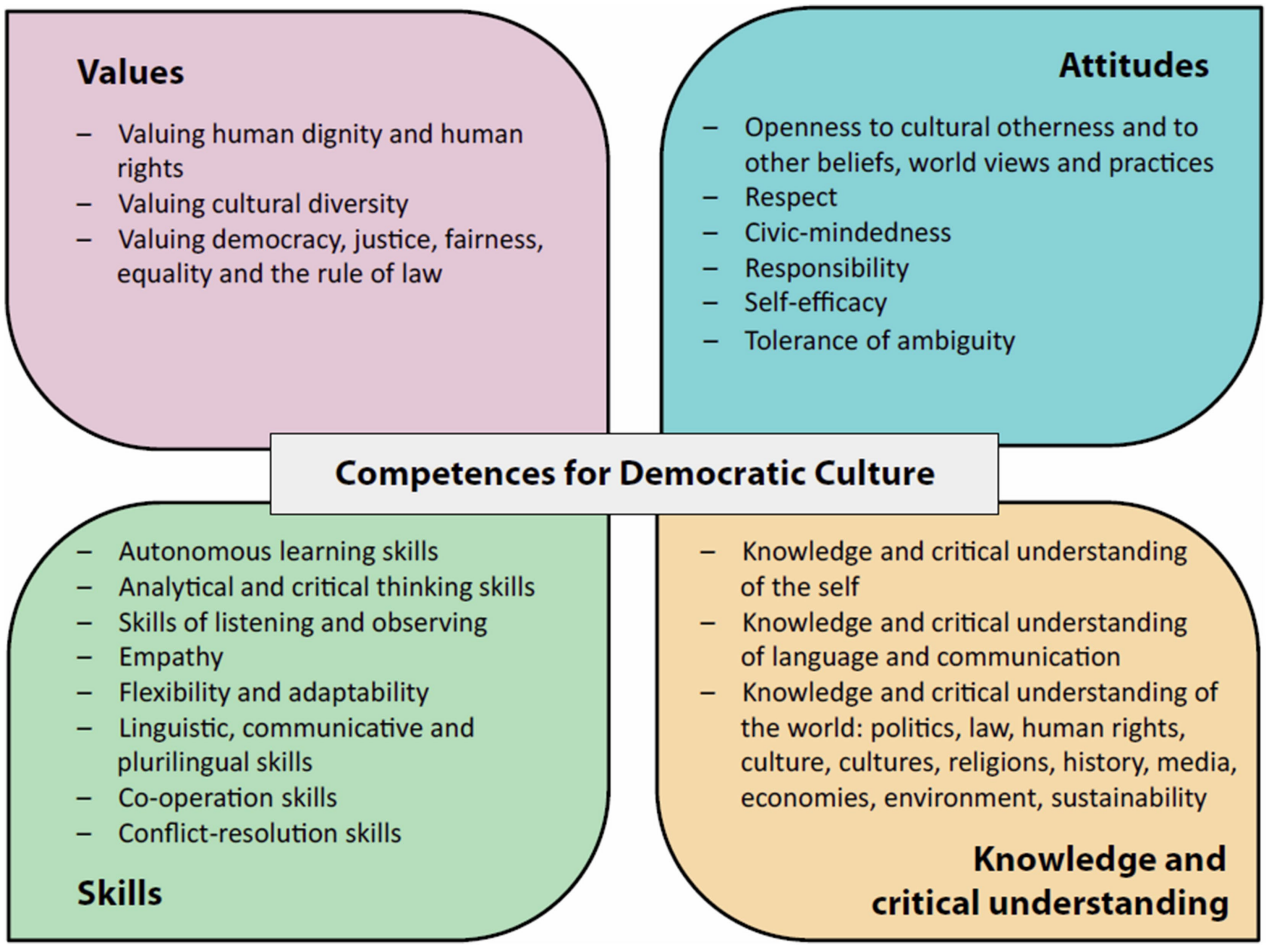
Reference framework of competences for democratic culture (RFCDC) [(COE), 2016, p. 38].

The KIDS4ALLL concept fits seamlessly into the overall mission of educational institutions, as complying with the vision set forth by UNESCO. This vision underscores the commitment to equipping learners with the skills and perspectives which are essential for active citizenship within a democratic society. The Reference Framework of Competences for Democratic Culture (RFCDC) further emphasizes the significance of this commitment, acknowledging its pivotal role in addressing both current and future societal challenges (COE, 2023b, p. 2) (Figure 1).

The RFCDC places the civic mission of education at its core, KIDS4ALLL puts this principle in place. Through its design, its method and its contents, KIDS4ALLL plays a vital role in shaping learners into active and engaged citizens as well as giving teacher’s and educator’s inspiration to be agents of change, contributing significantly to the ongoing development of a democratic society.

In the next chapters we see, how this initiative was received and what factors have influenced its impact during the pilot phase.

## 3 Methodology applied throughout the pilot-phase

The KIDS4ALLL project is designed in two main phases. The first one aims at the creation of the learning environment, and the second one to its testing and consequent improvement. This article focuses on its second phase: the pilot phase has been implemented across formal, non-formal, and informal institutions in nine countries (including three non-EU countries), ultimately reaching approximately 1,000 students. The countries involved are diverse, though share an enduring attachment to the teacher-centered pedagogical approach.

The data presented in this article have been collected in state schools at the elementary, middle and high school levels in Turkey. For each school level, one class was involved, comprising approximately 20–25 students. Diverse age groups were represented, with participants in elementary school falling between 8 and 11 years old (just the two upper classes were involved, attended also by older students), middle school students aged 11–14, and high school students ranging from 15 to 18. The Turkish education system for K-12 follows a compulsory and standardized structure, with defined curriculum guidelines for each grade level. Education in Turkey is compulsory for 12 years, known as the “4 + 4 + 4 system” governed by the Ministry of Education (MoNE) encompassing primary education (4 years; 6–10 years old), middle school (4 years; 11–14 years old), and high school (4 years; 15–18 years old) (Köseleci, 2015). The curriculum is systematized, and textbooks are standardized across the country, providing a consistent—and clearly delimited—educational experience for students (Kitchen et al., 2019). The MoNE plays a pivotal role in overseeing and regulating the curriculum, ensuring uniformity in content and teaching methodologies (OECD, 2020). The teaching approach emphasizes teacher-directed instruction and standardized assessments (Kızılçelik, 2015, p. 153). While recent educational reforms^1^ have aimed to introduce more student-centered and innovative teaching methods, the system still reflects a structured and centralized approach to education (İnce et al., 2022). It is essential to note that ongoing efforts are being made to adapt the Turkish education system to address the evolving needs of students and align with global educational trends (İnce et al., 2022; Özdemir, 2020).

The KIDS4ALLL project’s pilot phase in Turkey has been conducted in autumn 2022 and spring 2023 by a team of three researchers from the Koç University in three schools in the districts of Sarıyer and Şişli, including the Kurtuluş neighborhood, in Istanbul. These districts can be considered central urban spaces, characterized by diverse resident population groups. The selected schools were chosen based on the enrollment of students from low socioeconomic backgrounds, primarily with working-class parents, and migrant students.

The pilot phase has been introduced by two distinct sessions held at each school, engaging teachers and students separately. Comprehensive training sessions were conducted for participants slated to be involved in the upcoming pilot. The student training, lasting 2.5 h, initiated with a lively discussion on educational inequalities, followed by an exploration of KIDS4ALLL’s objectives. The eight key competences were then introduced through interactive games, culminating in the presentation of the buddy method. The concluding wrap-up provided an overview of the entire project, emphasizing its international character, highlighting that the same training and pilot were conducted in many partner countries. Interaction levels varied across educational tiers; high school students easily grasped and actively participated, middle school students required some assistance, while primary school students needed additional guidance and directives to navigate the training sessions.

The teacher training assumed paramount significance, given that teachers would implement the project and facilitate the students’ work with the learning units presented in the KIDS4ALLL handbook and platform. The training introduced the overall project and the buddy system, integrating a reflection on the pros and cons of existing pedagogical approaches. The training also introduced the learning method inherent to the project and the tools slated for adoption during the pilot. The pilot classes have been composed appointing students with different age groups and from different classrooms. Students were chosen through a process guided by the suggestions of principals and teachers, with an initial emphasis on pairing in so called buddy-teams migrant students with local counterparts. Subsequently, attention extended to matching bringing together students with different genders, ethnicity, and social skills. Likewise, teacher appointments were made on a voluntary basis. The buddy-teams have gone together through Learning Units (LU) which stimulate peer-learning around a specific topic. The LUs were available as print-outs, later as digital material on tablet and computers. In the middle and high school, almost every LU was led by different teachers, whereas in the elementary, it was three teachers who led together the sessions.

The pilot phase has been supported by qualitative empirical research. The data used in this paper have been collected through ethnographic observation and in-depth interviews with teachers and students in the selected schools. The ethnographic observation aimed to capture social and intercultural aspects of peer-to-peer and co-creational activities, intending to integrate these insights to improve the KIDS4ALLL learning environment. During the ethnographic observation, researchers observed all pilot classes involved. The analysis focused on examining the experiences of teachers and students during the pilot phase of the KIDS4ALLL project, exploring the challenges and opportunities encountered in implementing the project within the Turkish education system. Factors such as school-level, diversity, teacher involvement, and the effectiveness of the buddy system and of the LUs in promoting collaborative learning were considered. Observations were documented using observation sheets, capturing details of social interactions, engagement levels, and any challenges encountered during the pilot activities. Researchers also maintained field notes to record contextual information and personal reflections on the observed experiences. Out of the observation collected, a report has been written in which the experiences done by each researcher in the three contexts could be more deeply reflected. At the conclusion of the pilot phase in each class, both students and teachers were subjected to interviews covering the efficacy of the KIDS4ALLL learning method, their perspective on collaborative learning, and an overall assessment of the pilot experience. The interviews involved three teachers and four students from elementary schools, six teachers and four students from middle schools, and three teachers and five students from high schools. This multimodal research approach ensures a comprehensive understanding of the impact of the pilot program across different educational levels and facilitates triangulation of data from multiple perspectives. The collected data underwent qualitative analysis using thematic coding techniques. Transcripts of interviews and observational notes were coded and themes and patterns were identified, allowing for a nuanced understanding of the data. The triangulation of the data enhance the validity of the findings.

In adherence to ethical guidelines, this pilot study involving K-12 level students underwent rigorous scrutiny by the ethics committee, and all participant data has been meticulously anonymized to safeguard the confidentiality and privacy of the individuals involved.

## 4 Results

### 4.1 The physical space and the infrastructure

The physical space proved paramount in shaping the learning experience in all school settings.

In the elementary school, the learning activities unfolded in versatile spaces such as the school library and occasionally in the school garden. This deliberate departure from the traditional classroom setting aimed to create a distinct environment for students. Seating arrangements at roundtables facilitated a collaborative atmosphere, initially supported by handbook activities and copied pages. However, with the introduction of computers, a shift occurred—these devices were shared among peers and the roundtable format was changed to a rectangular combination of four desks where two students shared a computer and were seated facing each other. This transformation presented a primary challenge for teachers as it required effective classroom management and personalized assistance for each child.

In the middle school, the constraint of physical space has been an important challenge: its inadequacy posed challenges to its implementation, making more difficult for students to concentrate and collaborate. The designated computer room, essential for the pilot program, remained elusive at the project’s inception. This compelled teachers to resort to printed photocopies of the learning units as in the handbook until the computers were ready for use. The initial absence of digital resources presented a setback, impacting student interest and diminishing the excitement typically associated with the features offered by the KIDS4ALLL platform. The integration of computers into classrooms required infrastructure adjustments.

At the high school level, the pilot activities unfolded in a dedicated computer room arranged for students to work in buddy-pairs. This spacious room, adorned with expansive windows, offered both comfort and a welcoming ambient for students engaging in the pilot activities. The physical setting had been prearranged to keep students seated in pairs, with combined desks creating a comfortable gap between buddy groups. The room accommodated seven desks on the left and an additional seven on the right, with additional desks available for the teacher during lectures and observers. The intentional design of the physical space at the high school exemplifies a conducive environment, fostering collaborative learning and enhancing the overall pilot experience for students.

Due to certain restrictions imposed by Turkish state schools on internet connections for specific platforms to ensure student protection, some content despite being on open-access platforms, such as the Scratch platform that offers a free coding community for kids, were inaccessible via the school internet. According to teachers, the site in question had been utilized back in 2017, but its access was hindered, allegedly due to a question in the preface that asked for the participant’s gender. While some schools find solution to overcome this obstacle, others could not and substituted the learning unit with an alternative one.

### 4.2 Buddy-system

In the initial phase of the pilot activity, students’ initial hesitations underscored the novelty of the learning paradigm, embodying the inherent newsworthiness of this educational transition. In each level of schools, the hesitations varied.

In the high school, the first encounters witnessed hesitations as students were tasked with pairing up with their pre-assigned buddies. Notably, some students expressed a strong preference for altering their designated buddies in favor of individuals they were already acquainted with. However, the instructor did not permit this, although students were allowed to choose seats either at the back or front desks of their peers who were close to them. This inclination toward familiarity paralleled the discontent visible on their faces, reminiscent of the discontent often observed when students are required to change seats during regular class times due to a new seating arrangement. The resistance to change was palpable, emphasizing their desire for proximity to friends they already knew in the face of a shift in established arrangements. This visible discontent disappeared after a few meetings, and gradually, many of them started to develop relationships and collaborated to complete the learning units. However, two to three-buddy couples were more resilient to opening up. They either ended up changing peers or interacted with highly limited communication.

In the involved middle school, instances of student disruptions were relatively common: buddies were either talking to one another on different topics or including other buddy couples in their conversations. On certain occasions, these disruptions impeded the smooth presentation of buddy work to the entire class. Noteworthy early occurrences included a few students expressing a desire to change their assigned buddies, hinting at underlying tense social dynamics among students that were not immediately apparent.

In the participating elementary school, they were more obedient in sitting next to their assigned buddies and did not signal a direct discontent for the pairs they were assigned to, except if they were Arabic-speaking students, as they had difficulty understanding the materials. The Arabic translation of the contents was not ready yet. When the level of discontent was visible, the teacher intervened to encourage mutual participation to complete the tasks. Yet, the teachers did not allow any change of pairs, and students respected this authority. However, the consequence arose in the form of discontent and a lack of interest to participate. Especially, the migrant children encountered specific difficulties in comprehending and executing the assigned tasks. Despite teachers’ efforts to minimize interruptions during the students’ work, their active involvement was often necessary to clarify instructions and guide the children through their activities. In general, the constant stream of questions posed by the young students posed a significant challenge to the implementation of the buddy method and therefore teachers resorted to more traditional teaching methods.

The language proficiency of students in middle and high schools surpassed that of primary school students, including migrant students who exhibited fluency in Turkish. Given their ease in understanding and writing in Turkish, a language barrier was non-existent, requiring no additional explanations. Instead, the diverse cultural backgrounds contributed to bringing fresh perspectives into the classroom setting. The development of the students’ agency displayed in the original contributions which the different buddy-teams brought up.

The active participation of nearly two-thirds of the participants in the secondary school and in the high-school demonstrated a positive reception of the new method. The challenges encountered by the remaining one-third encapsulated the complexities inherent in managing a new system which gives students the chance to display their own agency. The sense of uncertainty was prevailing among students in the initial pilot activities, possibly stemming from the significant difference between the pilot and their own educational experiences. The unfamiliarity with everyone involved, coupled with the entirely new materials and format, contributed to a sense of novelty and required adjustment, stimulating students to learn to join the educational transition.

### 4.3 Teachers as facilitators

Amidst these varied responses, the pivotal role of teachers emerged prominently during the initial stage of the pilot. Depending on the teachers’ abilities, their influence either skillfully navigated the challenges inherent in the pilot, contributing to its success, or inadvertently prolonged the period of adjustment. The KIDS4ALLL pilot activities featured a range of didactic styles adopted by educators. These styles encompassed the use of cultural references, flexibility in teaching approaches, inclusivity, and a balance between formal and informal methods. Student engagement was influenced by the relevance of content and educators’ ability to adapt to students’ needs and interests. These observations highlight the importance of tailoring teaching styles to student demographics and the significance of engaging content in promoting active participation.

Moreover, as an intermediary, the teacher’s role in fostering dialogue took central stage in managing the diverse dynamics within buddy pairs. When teachers encouraged students to share their thoughts and knowledge with their buddy, support their understanding or added new team members to the group in order to stimulate the exchange, they contributed to active participation through giving the adequate support and adapting the concept to reality. This exemplifies a newsworthy instance of effective pedagogical facilitation. Conversely, the voluntary nature of participation coupled with the perceived extra workload without compensation, presented challenges. Some teachers struggled to find time for the pilot within their already hectic schedules.

In each school, they selected learning units which could be implemented within a single meeting. At the elementary school level, teachers diligently followed the organizational guidelines provided at the commencement of each unit. However, the young age of the students posed a unique challenge, as they tended to disengage toward the end of the unit, particularly during the final task of creating a postcard, a task connected to a reflection of their learning gains obtained through the KIDS4ALLL activities on the topic. In response, teachers made adaptive efforts, shortening certain activities to ensure that children had sufficient energy and concentration to finalize their learning journey with the foreseen reflection task. Though, as teachers found themselves needing to delve into more detailed explanations for younger students, especially in non-STEM areas where certain concepts were unfamiliar, such as “active citizenship,” they intervened to provide explanations from their perspectives. In some instances, they openly referenced students’ nationalities in classrooms or explicitly highlighted Turkish identity, creating separation. When non-Turkish students sought understanding, instead of employing a diverse approach, some teachers exhibited fatigue in re-explaining concepts to them. These signals or open statements conveyed mixed messages to the class, contradicting the overall aim of the project.

In the middle school, some teachers faced scheduling constraints that limited the implementation of learning units to once or twice. However, an encouraging aspect arose through the pre-existing familiarity between teachers and students, fostering a more congenial atmosphere in the classroom. Nonetheless, the adaptation to the buddy teaching method presented a challenge for certain educators, who found it difficult to diverge from traditional teaching methodologies. Some of them showed reluctance to adhere strictly to the provided instructions, opting instead to carry out the learning units in a manner that involved adjustments favoring one-sided didactic teaching with limited interactive elements. This presented considerable hurdles to managing student engagement and enthusiasm.

Shifting to the high school level, at the beginning of pilot session, the teacher assumed a more prominent role in guiding students, a necessary measure given the initial hesitancy displayed by students. Since each learning unit begins with an introduction delivered by the teacher, it then transitions to buddy teams to engage in related topics, including discussions, small tasks, or creative components, which they complete within a designated time frame. Following this, the teacher-led component briefly shifts the focus before once again providing time for buddy couples to collaborate on the topic. This structured approach allowed teachers to smoothly guide students through the pilot program, aiding in their understanding of the underlying logic and gradually leading to improved student performance. While this initially limited student interactions, it ultimately played a pivotal role in fostering group cohesion and cultivating enthusiasm within the class. Yet, the role of the teacher in the high school setting proved instrumental in navigating the transitional phase, acting as a facilitator to ease students’ adjustment to the novel learning method and techniques. Notably, high school teachers demonstrated a genuine interest in the pilot. The confluence of teacher dedication, active participation, and a supportive learning environment played a pivotal role in creating a conducive atmosphere for effective learning and adaptation to the innovative educational approach.

### 4.4 Students as active participants

After the initial phase, the evolving dynamics witnessed a shift in the attitude showed by most of them, indicating a growing comfort and familiarity with the pilot and their fellow classmates. As the dialogic and collaborative approach became familiar, an expansive environment for active student collaboration and participation developed, while most learners exhibited a heightened proficiency in leveraging this opportunity. Nevertheless, some students—not used to have such a free space in regular classes—used the time at disposal as it was spare time and were not able to benefit from it as they could have. Still, this progressive phase witnesses students not only acclimating to the complexities of the novel learning method but also actively engaging in critical thinking to evaluate its merits in comparison to established learning techniques.

This is particularly true for students in the high school. During the activities with their assigned buddies, the students involved began to foster deeper connections with each other. As the class familiarized with the pilot’s format, a growing interest emerged among certain students who contemplated having a say in the selection of content preferences. A divergence in interest surfaced, with students expressing a preference for non-STEM areas, particularly in subjects like multilingualism, cultural awareness and expression, and literacy skills. The learning units emphasizing these themes received high praise from students, fostering enthusiasm for collaborative activities, increased dialogue, and heightened creativity. This inclination was partly rooted in the perceived ease of non-STEM content for high school students, coupled with the desire to break away from the familiarity of STEM-related content. Furthermore, STEM contents are strongly connected with a true/false logic, leaving limited room for interactivity, dialogue, and sharing, which are the components that students enjoyed throughout the pilot. They seemed to connect the STEM contents to conventional teaching methodologies as adopted in their classrooms.

This underscores the challenge of crafting equally engaging contents within the STEM domain. For example, the “What is a Volcano?” unit in the STEM category failed to attract widespread interest among students, except for the creative aspect when they explored volcanoes in their own country. Conversely, the learning unit on “Intercultural dimension in music” proved highly enjoyable, with students actively engaging in creating non-instrumental music, showcasing increased interaction and enjoyment.

As the pilot progressed and the mandatory contents were completed, high school students asked whether they could choose one or two extra learning units. As a result, they selected two learning units from non-STEM areas. Their heightened level of interaction and participation in these specific units stemmed from the fact that they were based on the students’ decisions and focused on topics that genuinely interested them. One selected learning unit belonged to the “Personal, social, and learning-to-learn” category, while the other was in the “Multilingual” skills category. This marked a significant milestone where high school students, having fully comprehended the platform and its logic, demonstrated independence in declaring their preferences, subsequently becoming more creative and productive in topics of their choice. The intervention by the students, requesting to select their own topics of interest, signaled their development of critical thinking. The observed enthusiasm in their eyes while exploring which learning units to choose also indicated how they developed a path of self-discovery through the pilot process.

Yet, high school students enjoyed distinct advantages, feeling empowered to contribute actively rather than merely acting as consumers in the pilot program. This shift reflects a transformative aspect of their participation, as they evolve from passive recipients to active contributors, taking responsibility for their own learning experience and the one of their buddy.

However, this newfound interest was not universal among all high school students. A subset chose to use the allocated extracurricular time for socializing or feigned participation, opting to use it as free time rather than actively participating in the learning units with their assigned partners. Notably, approximately one-third of the group exhibited disinterest. This subset of disinterested students often simulated interest only when approached by the teacher. With distractions readily available in the form of phones and computers, this group of high school students found it easy to disengage when uninterested or bored with the content, exploiting the voluntary nature of participation. This specific group, feigning interest, covertly engaged in conversations or indulged in phone activities during interactive learning periods. In certain instances, a few students used excuses to go shortly out of the class but returned to the pilot class much later. Alternatively, if it was nearing lunchtime, some sought excuses and never returned, likely joining their friends for lunch.

Unlike the high school, where the progressive element remained consistent, the middle and elementary schools witnessed the gradual disappearance of both hesitation and enthusiasm initially displayed by students at the onset of the pilot. As students grasped the logic and structure of the program, they adopted a more relaxed stance. However, the initial allure and attraction that characterized the early phases of the pilot seemed to diminish. Middle school students, engrossed in high school exam preparations and essential classes, experienced a decline in concentration, while elementary school students exhibited noticeably lower engagement compared to other groups. Consequently, some students chose to attend their regular classes instead. Meanwhile, in the elementary school, a more relaxed atmosphere prevailed among students, but teachers found themselves consistently providing additional support to the buddy teams.

## 5 The crossroad is a roundabout: how to succeed in taking the new route

The article explores the transformative potential of education, focusing on insights from the KIDS4ALLL project, highlighting the need for a shift toward critical thinking, creativity, and inclusivity. The observation of teachers and students dealing with the new learning method and the corroboration of the perception of the researchers with the thoughts expressed by the teachers in the interviews shed light both on the chances and on the challenges of innovating education through the introduction of strategies promoting a participatory pedagogy within an entrenched system. The key findings can be summarized as follows: (1) the physical space influenced learning experiences across different school levels, emphasizing challenges and opportunities; (2) the buddy-system implementation varied across schools, with resistance to change observed but gradually overcome; (3) teachers played a pivotal role as facilitators, adapting teaching styles to promote student engagement and participation; (4) students exhibited evolving attitudes toward the pilot program, with some showing increased interest in non-STEM subjects. In terms of comparison, high school students, empowered to choose freely the learning unit, demonstrated critical thinking and active participation, though some remained disinterested. Whereas, middle and elementary school students showed diminishing enthusiasm over time, requiring ongoing support from teachers. Overall, the article underscores the complexities of educational transformation and the importance of teacher-student dynamics in fostering inclusive and impactful learning environments.

Furthermore, the analysis has let emerge five key factors that have influenced the way KIDS4ALLL was experienced as well as its possible impact on teachers and students, on their pedagogical relationship as well as on the design of future learning processes.

First of all the perception of this experimental moment as a parenthesis in a regular continuum. The fact that the participants of the pilot were cognizant of the temporary nature of the project introduced a distinctive challenge during the implementation process. Students grapple with the understanding that the transformative aspects introduced may not sustain in the long term. This realization prompts both teachers and students to hesitate in fully embracing the new emancipatory methodology, leading them to seek comfort in the familiar certainties of the traditional educational setting. The rapid transformation presented during the pilot phase thus places individuals in a transitional state where the allure of the old system competes with the potential benefits of the innovative approaches. Moreover, not just the perception of it as temporary but the fact that the pilot really had a temporary nature and was inserted in rigid, unchanged and seemingly unquestionable structures and routines impeded genuine engagement. The fact, for instance, that participation was not going to be graded—that means officially not recognized in the ruling system as worthy—made some students view it as an extended break, skipping part of the classes to hang out with friends or spending time surfing online rather than using it for self-development. On the teachers’ side, the voluntary nature of participation, coupled with the perceived extra workload without compensation, presented challenges, diminishing their motivation. As a result, some teachers struggled greatly to find time for the pilot within their already hectic schedules.

Second, the unfamiliarity with participatory peer-learning generally hindered the full grasp of the pilot’s objectives, leading to fall back into the traditional one-way teaching style or to the desire to complete the task quickly without questioning their purpose. Though following the initial phase of the pilot, students developed stronger affiliations with the program, its content, and their assigned buddies, and above all in high school most of them learned to learn with and from each other. Students’ affinity for the teacher and adherence to the teacher’s directives influenced students’ participation in the pilot. In situations with limited interaction among peers, the approach teachers took in guiding hesitant buddy couples into pilot activities, such as engaging them in dialogue or intervening as a mediator to facilitate communication among peers, significantly contributed to enhancing student interest and engagement. This underscores the importance of the ability of the teachers to understand themselves as facilitators and to perform it in a way which includes all students, fostering equal participation and positive peer-learning interactions. This project, aiming explicitly at the collaboration between migrant students and native ones shed light also on the challenges of teaching in a multilingual and diverse setting. Beyond language related skills, there is a whole set of competences which both teachers and students need to have, in order to facilitate and join participative processes, though precisely by engaging in the process they can develop them, albeit initial difficulties: those who have embraced the challenge and persevered found enjoyment and quickly developed new skills.

Third, the teacher’s openness in guiding and supporting students through this transformative educational experience played a crucial role in shaping the trajectory of the pilot, emphasizing the significant impact of pedagogical leadership on the successful implementation of innovative learning methods. It has emerged that the overall success of the learning process depends on the teachers’ engagement. More specifically, the teacher’s pivotal role as a facilitator during this transition became a noteworthy element of this educational shift, presenting a compelling narrative of adaptation in the face of change. In contrast to the prevailing methodological approach, where interactions occur individually between the teacher and each student, the hierarchical boundaries inherent in the existing system undergo a transformation, adopting a student–student format. The steadfast adherence of some teachers to systematic and structured teaching methods within the pilot program underscored the inherent challenges associated with inducing changes in entrenched pedagogical approaches. The reluctance of several teachers to embrace the new teaching approach highlighted the formidable task educators face when requested to alter established normalities, which norm the pedagogical action both reinforced by regulations, fixed processes and rigid structures and by the habit itself which limits the perception of one’s own freedom and the imagination of possible alternatives.

Fourth, the physical environment—meaning the type of space at disposal, its design and the digital infrastructure—had a strong impact on the learning experience. Inadequate physical space hindered participation of the buddy-teams and their concentration, while a conducive physical environment tailored to support the pilot’s execution significantly contributed to its overall success and implementation. It is worth to point out that the level of comfort perceived by the students could be observed and related to the different elements composing the settings. While the space influenced the quality of the interaction, the option of using or not using the platform had an impact on the motivation of the students. The excitement observed in relation to the use of multimedia material opens up a reflection on the importance of consciously integrating digital media in education, as to speak the language of the new generation. Furthermore, the fact that at the time of the pilot the Arabic translation was not yet at disposal nor was given the possibility of using translation apps shows how their integration in daily school life could greatly facilitate learning through improved understanding, active participation and collaboration while at the same time fighting exclusion and isolation.

Fifth, the quality of the piloted educational innovation concept is a core condition for its success. The analysis has revealed for instance that the level of engagement varied in relation to the different contents: while some learning units sparked curiosity, opened dialogue and collaboration, others were perceived as uninteresting, resulting in disengagement and individual work instead of teamwork. While the contents could be evaluated according to a variety of parameters—such as usability, esthetics, relevance of the topic—which might change from context to context and from user to user, the buddy-system as a learning method has proven to foster dialogue among students across different grades. The project has provided a platform for students to build new friendships and engage in dialogues that were previously uncommon. Doing so, students stepped out of the traditional learning patterns, experiencing an alternative teaching method and actively becoming a part of it. As mentioned above, the adaptation from a vertical to a horizontal orientation necessitated a period of adjustment at the commencement of the pilot. The questioning of established norms, wherein students engaged collaboratively with their peers rather than solely with the teacher led them to stimulate their own competences for democratic culture. This shift has not only impacted the dynamics within the classroom but also has initiated in some contexts a recalibration of power structures, challenging traditional hierarchies and fostering a more egalitarian approach to knowledge acquisition. This underscores the transformative potential of KIDS4ALLL.

This study has shown that when students are given the opportunity to break free from established hierarchies, they will do so, although they need a system that recognizes their commitment, teachers who support them by facilitating their learning process, and an environment that is appropriate and inviting. As teachers have the power to promote change, the KIDS4ALLL learning environment has a special section dedicated to them. There they can train their dialogic and intercultural skills, their ability to initiate and facilitate collaborative learning processes, as well as their global competence and socio-emotional management. Further research is needed to evaluate the impact of KIDS4ALLL on making teachers and educators agents of change in different contexts.

## Data Availability

The original contributions presented in the study are included in the article/supplementary material, further inquiries can be directed to the corresponding author.

## Footnotes

^1^Turkey’s Education Vision for 2023 outlines strategic objectives for inclusive and equality-based education, with the publication featuring the slogan “Happy Children, Strong Children” by the Ministry of National Education (MoNE). Additionally, significant steps were taken by the MoNE to revitalize educational programs, aiming to instill universal values such as justice, equality, peace, friendship, respect, and love across various disciplines, as highlighted in The 2nd Voluntary National Review Report of Turkey (2019). The national curricula, revised in 2018, were updated to align with the principles of sustainable development, global citizenship, and the integration of 21st-century skills (see İnce et al., 2022).
